# The invariant phenylalanine of precursor proteins discloses the importance of Omp85 for protein translocation into cyanelles

**DOI:** 10.1186/1471-2148-7-236

**Published:** 2007-11-28

**Authors:** Tobias Wunder, Roman Martin, Wolfgang Löffelhardt, Enrico Schleiff, Jürgen M Steiner

**Affiliations:** 1 Ludwig-Maximilians-Universität Munich, Department of Biology I, VW-Research Group, Menzinger Str. 67, 80638 Munich, Germany; 2 Max F. Perutz Laboratories, University of Vienna, Department of Biochemistry, 1030 Vienna, Austria; 3 JWGU Frankfurt am Main, Cluster of Excellence Macromolecular Complexes, Department of Biosciences, Max-von-Laue Str. 9, 60439 Frankfurt, Germany

## Abstract

**Background:**

Today it is widely accepted that plastids are of cyanobacterial origin. During their evolutionary integration into the metabolic and regulatory networks of the host cell the engulfed cyanobacteria lost their independency. This process was paralleled by a massive gene transfer from symbiont to the host nucleus challenging the development of a retrograde protein translocation system to ensure plastid functionality. Such a system includes specific targeting signals of the proteins needed for the function of the plastid and membrane-bound machineries performing the transfer of these proteins across the envelope membranes. At present, most information on protein translocation is obtained by the analysis of land plants. However, the analysis of protein import into the primitive plastids of glaucocystophyte algae, revealed distinct features placing this system as a tool to understand the evolutionary development of translocation systems. Here, bacterial outer membrane proteins of the Omp85 family have recently been discussed as evolutionary seeds for the development of translocation systems.

**Results:**

To further explore the initial mode of protein translocation, the observed phenylalanine dependence for protein translocation into glaucophyte plastids was pursued in detail. We document that indeed the phenylalanine has an impact on both, lipid binding and binding to proteoliposomes hosting an Omp85 homologue. Comparison to established import experiments, however, unveiled a major importance of the phenylalanine for recognition by Omp85. This finding is placed into the context of the evolutionary development of the plastid translocon.

**Conclusion:**

The phenylalanine in the N-terminal domain signs as a prerequisite for protein translocation across the outer membrane assisted by a "primitive" translocon. This amino acid appears to be optimized for specifically targeting the Omp85 protein without enforcing aggregation on the membrane surface. The phenylalanine has subsequently been lost in the transit sequence, but can be found at the C-terminal position of the translocating pore. Thereby, the current hypothesis of Omp85 being the prokaryotic contribution to the ancestral Toc translocon can be supported.

## Background

The plastids of glaucophytes, rhodophytes, green algae and higher plants are surrounded by an envelope consisting of two membranes. These "primary" plastids are thought to have originated from a single primary endosymbiotic event [e.g. [1]]. Cyanelles (muroplasts) are the peptidoglycan-armored plastids of glaucocystophyte algae which represent the first diverging phototrophic eukaryotes, on the earliest branch after initial endosymbiosis. Cyanelles can be envisaged as the closest cousins to free-living cyanobacteria among plastids [2-4]. The unique murein layer serving as "organelle wall" is modified through amidation of the free C-1 carboxylic group of the D-isoglutamyl moiety with N-acetylputrescine [5], which is unusual in the eubacterial kingdom and reduces the negative net charge of the murein layer that might interfere with protein import. Cyanelle (and likely the red algal) protein import machineries in plastids should be considered as prototypes of translocation systems that later underwent substantial modifications. These primitive translocons strictly require phenylalanine in the N-terminal domain of the transit peptide, even when they fulfil their function in a secondary plastid derived from a red alga [6]. A key event for transition of the "primordial plastids" to the chloroplasts of green algae and higher plants is the gain of (additional?) receptors in the Toc complex conferring less stringent and overlapping specificities finally leading to dispensability of the once crucial phenylalanine [7].

Recent reports confirmed the dependence of protein translocation across the outer membrane of cyanelles or the third outermost membrane of plastids derived from secondary endosymbiosis through a red alga, on the presence of a phenylalanine within the transit sequence [8-10]. In the absence of import experiments with red algae, the similarity of the N-terminal phenylalanine motif of *Porphyra yezoensis *plastid precursor proteins [11] to their counterparts from *C. paradoxa *is striking. However, the translocation of precursor proteins across the outer membrane of higher plant and green algal chloroplasts does not show such dependence [7]. Thereby, the phenylalanine represents a key for the understanding of the development of translocation machinery and their receptors. Yet, one question has not been explored satisfactorily, namely, what the phenylalanine is needed for. Current models favour an interpretation where this amino acid interacts with the ancestral translocon built up by endosymbiont-derived Toc75 [7,12]. However, other models on the evolutionary development and mechanism of translocation focused on an initial specific interaction between the precursor protein and the organellar membrane [e.g. [13,14]]. We reconsidered both possibilities, since phenylalanine is an aromatic amino acid known to exhibit pronounced hydrophobic properties [15], and it is known to insert more deeply into the membrane than other less hydrophobic amino acids like alanine [16]. Furthermore, phenylalanine is one important component in "aromatic belts" demarcating the hydrophobic surface immersed in the lipid bilayer [17]. It is noteworthy that all bacterial outer membrane protein structures analyzed so far show an interaction between their ultimate amino- and carboxyterminal domains. In some cases this contact is closing the β-barrel structure completely (e.g. *E. coli *OmpC [18]) or in addition forming a hydrophobic interaction between a phenylalanine in the N-terminal domain and the C-terminal phenylalanine (e.g. *E. coli *OmpG [17]). These observations would favour a role of phenylalanine in membrane association of the cytosolic precursor as first contact with the cyanelle.

The importance of aromatic amino acids in protein-protein interactions or substrate recognition is manifold documented. For example, aromatic residues are found in the substrate-binding site of the AAA+ chaperone ClpB that are located at the central pore of the first AAA domain. These aromatic residues may act as a molecular clamp by binding and releasing substrates in a nucleotide-dependent manner [19]. Translocation of exposed segments enriched in aromatic residues (phenylalanine, tyrosine and tryptophane) of a protein aggregate by ClpB would lead to the continuous extraction of unfolded polypeptides from an aggregate. Therefore hydrophobic interactions between aromatic residues are a common principle of protein-protein cooperation. Furthermore, phenylalanine is reported to occupy prominent positions in the sequences of proteins targeted to the bacterial outer membrane [20], such as porins (C-terminal amino acid) and type IV pilins (N-terminal amino acid, after cleavage by prepilin peptidase). Recently it has been shown that the penultimate residue of bacterial outer membrane proteins is involved in the species specificity of Omp85 recognition [21]. Interestingly, cyanobacterial outer membrane proteins show the C-terminal consensus sequence FxF [22]. Thereby, it is tempting to speculate that a phenylalanine (an aromatic) residue is important for the interaction of precursor proteins with the Toc translocon of primitive plastids.

In here we describe the influence of the phenylalanine on the interaction with lipids and lipid surfaces represented by liposomes and proteoliposomes. The obtained specificity for proteoliposomes is subsequently discussed in the context of the two "initial receptor models" and the evolutionary development of the chloroplast translocon.

## Results

### The phenylalanine defines lipid specificities of pFNR

**Figure 1:**
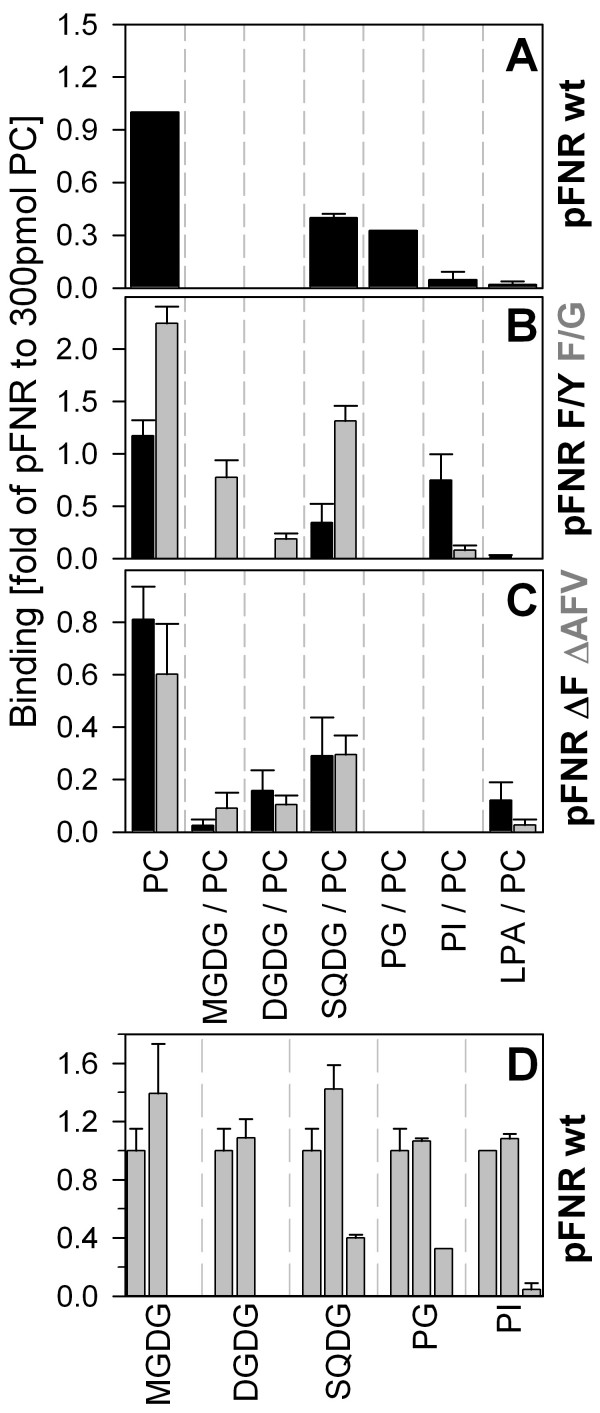
**Binding of Cyanophora pFNR and mutants to immobilized lipids**. (A) The wild type or mutant pFNR (B, C; bar colour indicated corresponds to label colour on the right site) were incubated with blots coated with 300 pmol of PC or 15 mol% PC and 85 mol% of the indicated lipid (see bottom of C). The binding was quantified, normalized to added protein and to the binding efficiency of wild type pFNR to 300 pmol PC. D) The same experiment as in A was performed using 100 mol% (left bar), 85 mol% (middle bar) or 15 mol% of PC (right bar) supplemented with the indicated lipid. In A-D) the average of at least three independent experiments and the standard deviation is shown. (DGDG, digalactosyldiacylglycerol; LPA, lipid A; MGDG, monogalactosyldiacylglycerol; SQDG, sulfoquinovosyldiacylglycerol; PC, phosphatidylcholine; PG, phosphatidylglycerol; PI, phosphatidylinositol).

We first analyzed the association of the precursor of the ferredoxin:NADP^+^-oxidoreductase (pFNR) from *Cyanophora *to lipids found in the membrane of chloroplasts isolated from higher plants [e.g. [13,14]]. Even though the lipid content of the outer membrane of cyanelles is not yet known, it is envisioned that loss of the lipopolysaccharides paralleled the endosymbiotic process [e.g. [23]] and that they were replaced by phosphatidylcholine and galactolipids [e.g. [24]]. Hence, the composition of the outer membrane of e.g. pea chloroplasts serves as a model for the lipid composition of the outer membrane of cyanelles. We used the well established lipid dot blot (Fat Western) analysis [25] to determine the interaction between precursor and specific lipids. The precursor protein interacts with phosphatidylcholine (PC) (Fig 1A). Interestingly, pFNR did not show an interaction with other neutral lipids (Fig 1A), but with the two charged lipids sulfoquinovosyldiacylglycerol (SQDG) and phosphatidylglycerol (PG), though to a reduced extent when compared to the interaction with PC (Fig 1A). We further controlled whether the lipids are capable of rejecting pFNR by mixing 15 or 85 mol% of the lipids to PC (Figure 1D). However, at the lower concentration of the added lipid the association of pFNR is comparable to its binding to PC (Fig 1D).

To explore the influence of the phenylalanine on this interaction, we used the two types of mutants previously generated [8]. In a first set, the phenylalanine was replaced either by a tyrosine or by glycine. In a second set, the phenylalanine itself or with the two neighbouring amino acids were removed. When binding of the mutants to lipids was analyzed we did obtain the highest affinity for PC as seen before for pFNR. However, some changes in lipid binding can be found. The most conservative mutant F→Y parallels the feature of wild type in terms of not interacting with mono – or digalactosyldiaclglycerol. (Fig 1B). It still binds to SQDG, but not to PG, suggesting that its interaction to SQDG is not driven by electrostatic interactions. In contrast, the exchange of the phenylalanine by glycine introduces marked alterations (Fig 1B) as this mutant shows no binding to PG but to all galactolipids. The two deletion mutants reveal a reduced but still recognizable binding to PC and SQDG, but not to the other lipids (Fig 1C). Hence, we obtained a chain of alterations of lipid specificities or binding capacities, which somewhat, but not fully parallel the import behaviour observed [8].

### The aromatic properties of the phenylalanine define the lipid specificity

**Figure 2:**
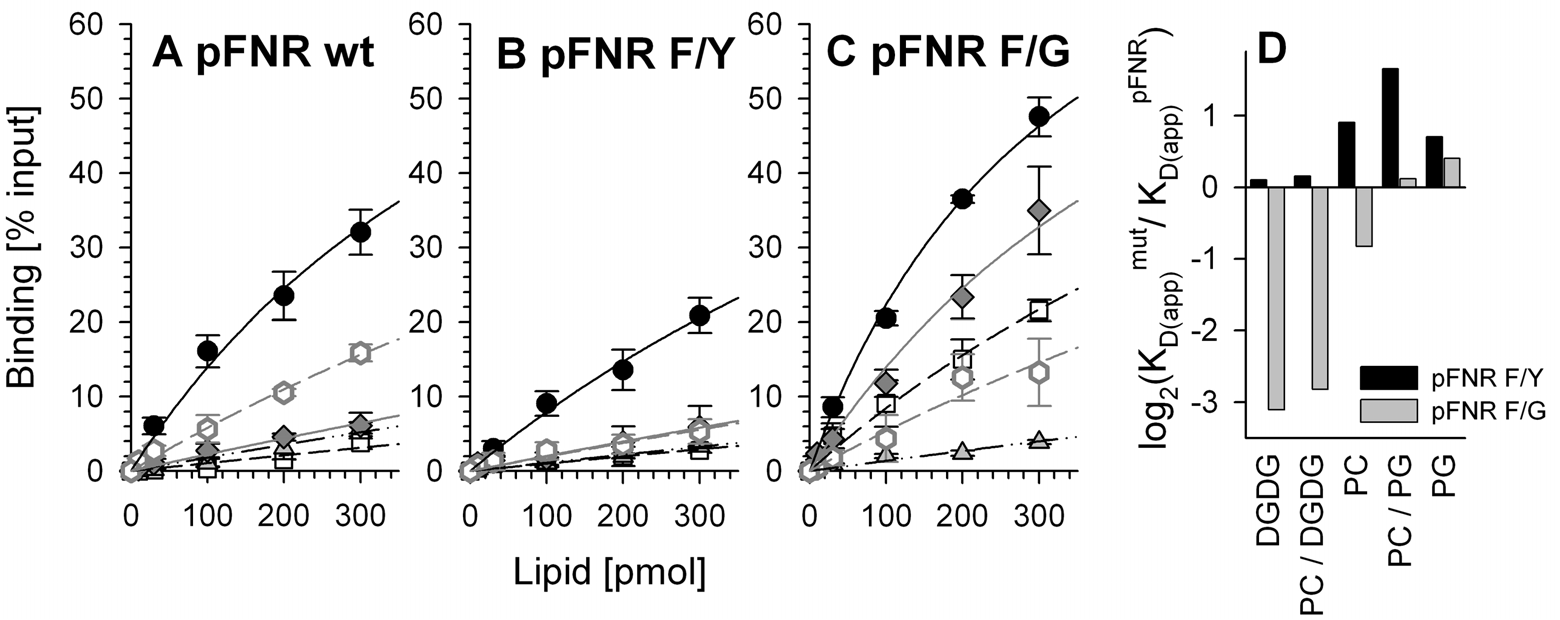
**Replacement of the aromatic amino acid changes lipid binding properties of pFNR**. The binding of pFNR (A), pFNR F→Y (B) and pFNR F→G (C) to indicated amounts of immobilized PC (black circle), DGDG (open square), PG (grey triangle), 85 mol% DGDG/15 mol% PC (grey diamond) or 85 mol% PG/15 mol% PC (open grey hexagon) was determined as described and binding normalized to the input is shown in percent. In A-C) the average of at least three independent experiments and the standard deviation is shown. Lines represent the least square fit analysis to equation 1. (D) The apparent dissociation constant for the association of pFNR F→Y (black) and pFNR F→G (grey) with the indicated lipids was calculated (E1) and normalized to the apparent dissociation constant of the wild type protein for the according lipid. The dual logarithm is shown.

To further support our findings we have focused on the comparison between wild type and the two mutants with amino acid exchange. These two mutants have differential import rates [8] and an altered lipid binding behaviour (Fig 1A, B). We analyzed the binding of the three proteins to PC, DGDG and PG as PG was found to be specifically recognized by the wild type precursor and DGDG specifically by the mutant with phenylalanine to glycine exchange (Fig 1). We were indeed able to confirm the highest binding capacity for all proteins to PC (Fig 2A–C) and the enhanced capacity for this association by the F→G mutant. Based on these experiments we were able to determine an apparent dissociation constant. To directly explore differential features the ratio between the apparent dissociation constants of the mutant and wild type for each lipid, was calculated. By this it becomes obvious that the conservative exchange from phenylalanine to tyrosine somewhat reduces the association with phospholipids, but does not effect the affinity for galactolipids (Fig 2A, B, D). In turn, the exchange of phenylalanine to glycine enhances the association to galactolipids or PC considerably, whereas the association to PG is somewhat reduced (Fig 2A, C, D). However, this reduction is not a result of the charge as pFNR F→G binds do SQDG better than wild type (Fig 1). Therefore, the exchange of phenylalanine to another aromatic amino acid only slightly affects binding by reducing the affinity to phospholipids but not altering the affinity for galactolipids, whereas removal of the aromatic amino acid at this position significantly enhances affinity for galactolipids without significant alterations of the affinity for phospholipids.

### Binding of pFNR to liposomes

**Figure 3:**
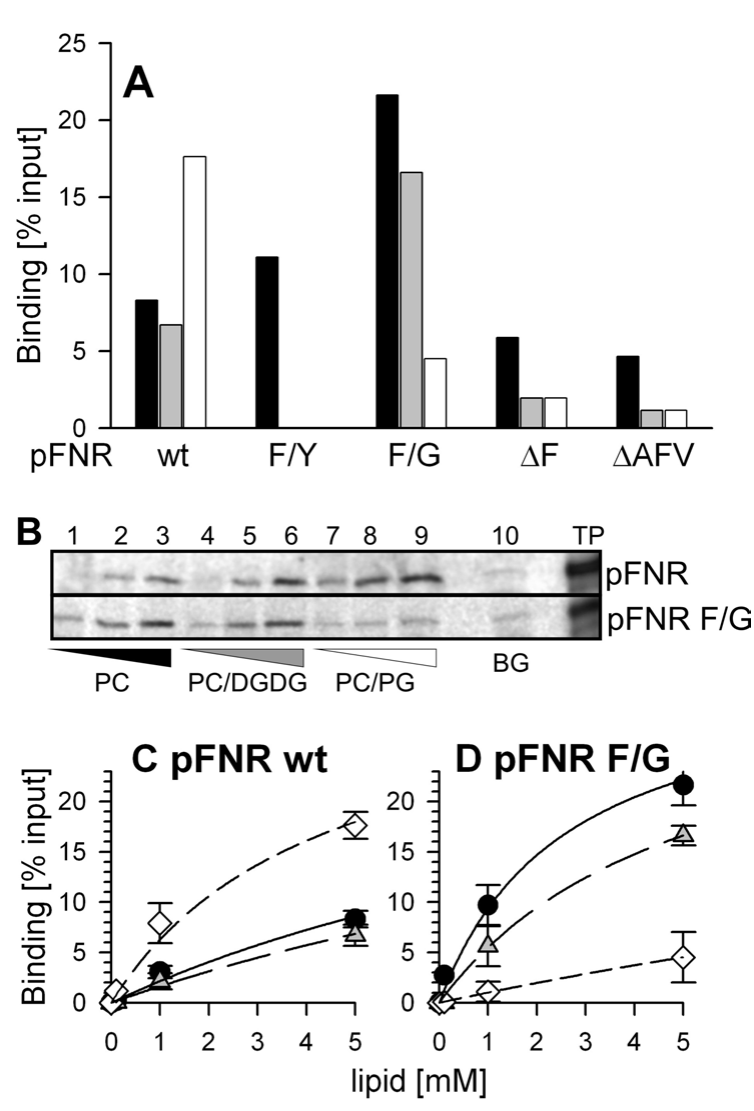
**Binding of pFNR and mutants to liposomes**. (A) 5 mM (lipids) of liposomes with a diameter of 200 nm generated as described [41] and composed of PC (black bar), 85 mol% PC and 15 mol% DGDG (grey bar) or 85 mol% PC and 15 mol% PG (white bar), were incubated with labelled precursor and binding analyzed as described. The binding is expressed as percent of the added precursor. Shown is the average of at least three independent measurements. (B) Labeled pFNR (top) and pFNR F→G (bottom) was incubated with 0.1 mM (lane 1, 4, 7), 1 mM (lane 2, 5, 8) and 5 mM (lane 3, 6, 9) liposomes composed of PC (lane 1–3), 85 mol% PC and 15 mol% DGDG (lane 4–6) or 85 mol% PC and 15 mol% PG (lane 7–9) and binding determined as described, Lane 10 represents radioactive protein processed in the absence of liposomes. (C, D) Experiments as shown in B) were quantified and binding to liposomes composed of PC (black circle), 15 mol% DGDG/85 mol% PC (grey triangle) or 15 mol% PG/85 mol% PC (white diamonds) are expressed as ratio to input. The average of at least three independent measurements is expressed.

So far we have determined the association of the proteins to immobilized lipids. To further support our findings and to establish a system to reconstitute the Toc75 like proteins, we have analyzed the association of the precursor proteins to liposomes (Fig 3). We were able to reproduce the findings with immobilized lipids in terms of the enhanced affinity of pFNR F→G for the liposome surface composed of PC or PC and DGDG, the loss of interaction with PG or DGDG containing surfaces by the F→Y replacement and the reduced affinity of the mutant with deleted phenylalanine (Fig 3A). However, we also obtained a difference when compared to the Fat Western analysis, as pFNR wt binds significantly better to liposomes containing 15 mol% PG than to liposomes composed of PC only (Fig 3A). We subsequently analyzed the binding in more detail by incubation of radioactive precursor proteins with different concentrations of liposomes (Fig 3B). The specificity of the interaction was controlled by analyzing the precipitation of the precursor in the absence of liposomes (lane 10). Quantification of these experimental data revealed a four to five fold lower apparent dissociation constant (according to equation E1) for the binding of pFNR to PC/PG liposomes than to PC or PC/DGDG liposomes (Fig 3C). For pFNR F→G we again obtained the highest efficiency for PC liposomes revealing an apparent dissociation constant, which is two times lower than the binding of the mutant to PC/DGDG liposomes or the wild type protein to PC. The mutant shows the lowest binding capacity for PC/PG liposomes. Here, the apparent dissociation constant is 10 times higher then for PC liposomes.

Summarizing, we document that the alteration of the phenylalanine modifies the lipid binding behavior of the precursor protein to lipids immobilized to a surface or present in liposomes. In both cases, deletion of the phenylalanine results in a reduction of the association (Fig 1C; 3A). The exchange of the phenylalanine to tyrosine somewhat changes the specificity for the lipids, but does not significantly change the affinity to phosphatidylcholine (Fig 1B; 2B, D; 3A). In contrast, exchange of phenylalanine to glycine on one hand enhances the association to phosphatidylcholine and changes the specificity of the protein from preferred interaction with phosphatidylglycerol to preferred interaction with galactolipids (Fig 1B; 2C, D; 3A, D). This discrepancy can not be explained by a difference of the hydrophobicity within the mutants. Considering the first seven amino acids, the wild type protein shows a mean hydrophobicity of 0.31 according to the Eisenberg scale [e.g. [26] and references therein], and the F → Y and F → G mutants a hydrophobicity of 0.22 and 0.24, respectively.

### Binding of cyanelle pFNR to Omp85 from Anabaena or Toc75 from pea

**Figure 4:**
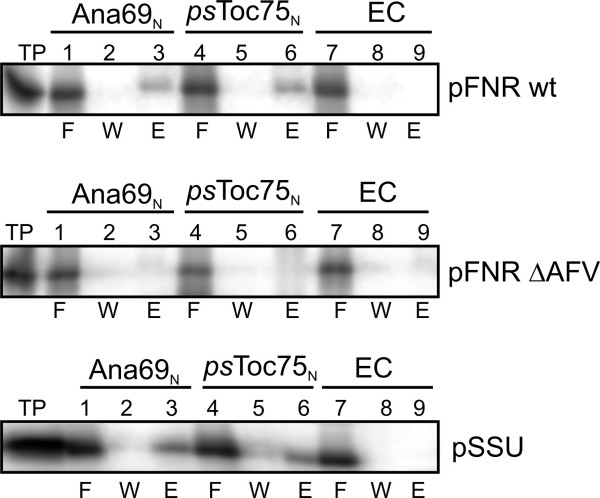
**Binding of pFNR to the precursor binding domains of Omp85-like proteins**. Radioactively labeled pFNR (top), pFNR ΔAFV (middle) or pSSU (bottom; lane 1, 100% input) was incubated with an Ni-agarose [28] affinity matrix without coating (empty column, EC, lane 7–9) or coated with the N-terminal domain of Alr2269 (Ana69_N_, lanes 1–3) or pea Toc75 (psToc75_N_, lanes 4–6). The flow through (F; lanes 1, 4, 7), material from the last wash step (W; lanes 2, 5, 8) and the eluate (E; lanes 3, 6, 9) was subjected to SDS-PAGE and binding visualized by autoradiography.

**Figure 5:**
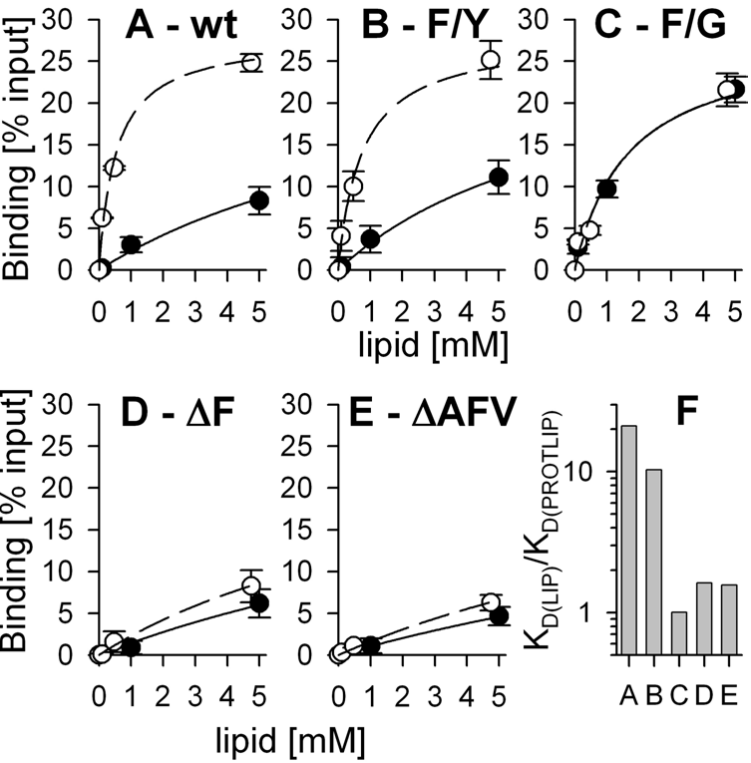
**Binding of pFNR and mutants to proteoliposomes**. pFNR (A), pFNR F→Y (B) and pFNR F→G (C), pFNR ΔF (D), or pFNR ΔAFV (E) were incubated with indicated concentrations of liposomes (black circles) or proteoliposomes (open circles) with a ratio of 1:2375 (protein to lipid, molar ratio). The binding was quantified as described and is expressed as percent of input of precursor protein. The data were fitted to equation 1. F) The ratio between the apparent dissociation constant determined for the binding to liposomes and for the binding to proteoliposomes is shown. The labelling of the x-axis is according to the sub-figure labelling.

**Figure 6:**
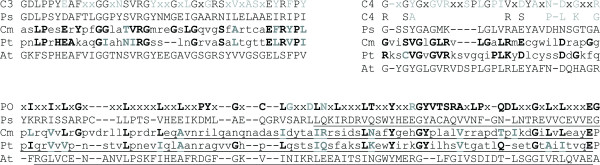
**Motifs in putative Omp85 proteins from *P. tricornutum *and *C. merolae***. Sequences from *P. tricornutum *(jgi|Phatr2|36936|fgenesh1_pg.C_chr_11000289, [43]) and *C. merolae *(CMJ202C, [44]) were found to share similarity with the Omp85 family. Shown is the alignment to three characteristic motifs of this family, the POTRA domain [36] as well as motifs 3 and 4 identified while analyzing a large class of polypeptide transporting β-barrel proteins [7]. Alignments were produced with MAFFT at standard setting using the L-INS-I strategy ([45, e.g. 46]) considering the consensus sequence POTRA (PO), consensus motif 3 (C3), consensus motif 4 (C4), the two identified sequences in *P. tricornutum *(Pt) and *C. merolae *(Cm), the sequence from Toc75 (GenBank:Q43715) found in *Pisum sativum *(Ps) and Toc75-V (TAIR:At5g19620) from *Arabidopsis thaliana *(At). The amino acids highly conserved in the motifs are shown in black, amino acids representing a preference but not a high conservation are shown in grey and positions with high variation with x. Amino acids within the two sequences identified matching the motif are highlighted in bold. Lower case amino acids on Cm and Pt sequence indicate positions either not conserved in the motif or in the sequence found. The region representing a POTRA domain identified while searching for conserved domains [47] is underlined.

Our results for the lipid binding do no parallel the previous observations while importing the respective constructs into pea chloroplasts or cyanelles [8]. We therefore determined the association of pFNR with two Omp85-like proteins from pea (Toc75) and *Anabaena *sp. PCC7120, because the latter protein encoded by alr2269 is thought to be related to the ancestral translocon [27,28]. pFNR binds to both proteins (Fig 4, top) with comparable efficiency to the precursor of the small subunit of ribulose-1,5-bisphosphate carboxylase/oxygenase (pSSU) from pea. However, no interaction to the matrix was obtained in the absence of a coating protein (lane 9) or using the mutant lacking the amino acids alanine, phenylalanine and valine (ΔAFV; Fig 4, middle row). Hence, the precursor protein is recognized by the two Omp85 family proteins, and the N-terminal portion of the transit peptide is responsible for this interaction. The results are also in line with the protein translocation into pea chloroplasts, where the deletion of the three amino acids causes a significant reduction. To further explore this interaction we studied it in a membrane environment. The Omp85-like protein from *Anabaena *sp. PCC7120 was reconstituted into liposomes composed of phosphatidylcholine. The binding of the wild type precursor to liposomes is significantly enhanced in the presence of the Omp85-like protein (Fig 5A). The apparent dissociation constant determined was about 20 fold reduced (Fig 5F). The exchange of phenylalanine to tyrosine somewhat reduced the affinity for the proteoliposomes (Fig 5B) resulting in a 9 fold reduction of the apparent dissociation constant (Fig 5F), which is half of that found for wild type pFNR. This observation parallels the decrease in *in vitro *import efficiency into cyanelles of this mutant when compared to wild type. The mutant carrying a glycine did not show an enhanced binding to proteoliposomes (Fig 5C, F). Interestingly, this precursor was also not imported into isolated cyanelles. In line, the binding to liposomes of the two deletion mutants, which are incompetent for import into cyanelles [8], was insignificantly enhanced when using proteoliposomes (Fig 6D, E), because the increase was in the error range of the experiment. Therefore, binding of the two deletion mutants and the mutant with a phenylalanine exchange to glycine did not show a significant dependence on the presence of the Omp85 homologue in the liposome which parallels the loss of import competence [8]. We can therefore postulate that the dependence of protein translocation into cyanelles on the presence of N-terminal phenylalanine is caused by Omp85 protein, most likely via its receptor domain.

## Discussion

Previous reports implicated a monogalactosyldiacylglycerol dependent interaction of the transit peptide of tobacco pSSU with liposomes [29] and a preference of the transit peptide of ferredoxin from *Silene pratensis *for 1,2-dioleoyl-sn-glycero-3-phosphoglycerol and sulfoquinovosyl-diacylglycerol [30]. These findings are in contrast to our observations for the ferredoxin:NADP^+^-oxidoreductase (pFNR) from *Cyanophora*, which binds strongest to phophatidylcholine (Fig 1, 2, 3). The exchange of phenylalanine to glycine within the cyanelle precursor causes the most significant alteration of lipid binding, especially with respect to the affinity and lipid preference. For this mutant we obtained almost no binding to lipid mixtures with phosphatidylglycerol, but an enhanced interaction with liposomes containing galactosyldiacylglycerol (Fig 1, 2, 3). Hence, our results suggest that exchange of the phenylalanine by a non aromatic amino acid renders the physiochemical properties of the transit peptide from *Cyanophora *closer to those found for transit peptides of organisms with chloroplasts *sensu stricto *[29-32].

However, our analysis revealed a picture of lipid binding significantly different to that observed during import of precursor proteins into cyanelles [8]. Hence, we have to conclude that lipid association alone does not account for the observed phenylalanine dependence of protein translocation into cyanelles. In turn, the efficiency of precursor binding to proteoliposomes clearly parallels the import efficiencies of the mutant precursors. Interestingly, psToc75, a member of the Omp85 family, discriminates between *C. paradoxa *pFNR with and without phenylalanine. However, the binding of pSSU from pea also documents that this does not mean a dependence on phenylalanine *per se *(because this precursor does not contain a phenylalanine in the transit peptide) but rather on so far not understood physicochemical features of the transit peptide. Nevertheless, this results points toward the presence of an Omp85 like protein in cyanelles functioning as receptor recognizing the phenylalanine present in the transit sequence. In line, Omp85 proteins are found to be involved in protein translocation across membranes or integration of outer membrane proteins of bacteria and endosymbiont-derived organelles [e.g. [12,33-35]]. Furthermore, in the diatom *Phaeodactylum tricornutum*, where translocation across the third outermost membrane is phenylalanine-dependent as well [9], a member of the Omp85 family can be identified by sequence similarity. This Omp85-like protein possesses a bipartite plastid targeting signal (with F as the first amino acid after the signal peptide cleavage site), displays a POTRA domain (Fig 6, [36]) and ends with the cyanobacterial C-terminal consensus (ADFDF). Thereby, the C-terminus differs from that found in Toc75 proteins of green algae and higher plants (GERF/Y), but is similar to that found in an Omp85 homologue in the red alga *Cyanidioschyzon merolae *(VDVSY). The two proteins from *P. tricornutum *and *C. merolae *do not display the N-terminal polyglycine stretch typical for the Toc75 proteins [37], but contain an LRGGG motif in the N-terminal region expected to be soluble. Additionally, the two identified sequences contain motifs 3 and 4 identified in the Omp85 family (Fig 6, [38]). Hence, these observations together with preliminary data from heterologous western blots (F. Yusa and J. Steiner, unpublished observation) support the existence of an Omp85 like protein in the outer membrane of cyanelles, which serves as receptor for the incoming precursor.

## Conclusion

Summarizing, the following model for Toc translocon evolution can be assumed from the presented data. Primordial transit sequences evolved with a phenylalanine for two reasons; at first this amino acid attenuates the interaction with the cyanelle surface containing mono- and digalactosyldiacylglycerol. Interestingly, previous studies determined that diacylglycerol was needed to interfere with spontaneous insertion of proteins into liposomes using an *E. coli in vitro *system [39]. In the absence of diacylglycerol spontaneous integration into phospholipids was observed even for multi-spanning membrane proteins. It was concluded that diacylglycerol seals the cytoplasmic membrane of *E. coli *against spontaneous insertion of hydrophobic proteins. Hence, the low affinity of pFNR for galactosyldiacylglycerol containing membranes might be essential to warrant the recognition by proteins present in the outer membrane. One such protein is Omp85 involved in outer membrane protein assembly in bacteria and predestined to interact with sequences composed like transit peptides [27]. In a primitive translocon, this receptor pore likely is the only candidate to interact with precursor proteins whereas in the chloroplast system a whole set of receptors is available for binding and able to overrule precursor-lipid affinities. Secondly, the phenylalanine reflects a match to properties of bacterial Omp85 proteins, which are thought to recognize OMPs via their C-termini enriched in aromatic amino acids [e.g. [20,21]]. In the course of evolution and paralleled by development of a more sophisticated translocation apparatus including cytosolic guidance complexes and regulatory receptor components like Toc34 and Toc159 [e.g. [40]], the importance of the N-terminal phenylalanine might have been gradually lost in the "green line", which allowed a more flexible and versatile (plastid type- and tissue-specific) regulation of the import process. Future research, especially on the translocon of cyanelles, will have to corroborate this model.

## Methods

### General methods

Construct generation, *in vitro *transcription and translation and protein expression and purification are described in [8,27,28]. Purified plant lipids were purchased from Nutfield Nurseries (Surrey, UK). Lipid A and fatty-acid free BSA was obtained from Sigma (Munich, G). The binding of precursor proteins to the N-terminal construct of psToc75 and alr2269 (anaOmp85) was analyzed as previously described [28].

### FAT blot assay

Lipids were diluted in chloroform and indicated amounts spotted onto PVDF membranes [25]. The membrane was subsequently saturated with 0,25% fatty-acid free BSA for 1 hour. Afterwards, 12,5 μl of the indicated translation product was diluted into 5 ml of 0,25% fatty-acid free BSA and 1 mM methionine and incubated for 1 hour at 20°C while rotating the blot. The blot was subsequently washed by 3 incubations with 1 mM methionine and 0,25% fatty-acid free BSA for at least 15 min, dried and binding quantified by phospho-imaging (Reader FLA 3000, Fuji-Film, Tokyo, Japan). The binding efficiency was determined by parallel detection of radioactivity in the added translation product using AIDA-Image Analyser software (Raytest, Isotopenmessgeräte GmbH, Steubenhard, G). In Figure 1, the binding was further normalized to the efficiency of the interaction between wild type pFNR and 300 pmol PC spotted to the PVDF membrane.

### Liposome generation and protein reconstitution

For binding analysis liposomes with a 200 nm diameter were produced as described [41] in 20 mM Tris/HCl, pH 7,4 and 300 mM sucrose and diluted to a final concentration of 10 mM lipids. For reconstitution 4 mg phosphatidylcholine were resuspended in 400 μl 20 mM Tris/HCl pH 7,4 and 400 mM sucrose and Mega 9 was added to a final concentration of 80 mM. Proteins (0,2 mg; in 5 M urea, 50 mM NaPi pH 6.8, 150 mM NaCl, 10 mM β-Mercaptoethanol, 500 mM Imidazol) were also incubated with Mega 9 (80 mM final), subsequently added to the lipid mixture and dialyzed over night against a 2000 fold excess of 20 mM Tris/HCl, pH 7,4; 200 mM sucrose. The proteoliposomes were pelleted for 10 min at 80.000 × g, resuspended in 20 mM Tris/HCl, pH 7,4; 200 mM sucrose and stored at -80°C. Before use, liposomes were thawed and sonicated for 10 sec. Protein reconstitution was controlled by western blot analysis (not shown).

### Liposome binding experiments

The protocol was adjusted according to [42]. Liposomes and proteoliposomes at indicated concentrations were incubated for 10 min at 20°C with 5 μl of the radioactive labelled precursor proteins in 20 mM Tris/HCl pH 7,4 and 100 mM NaCl (100μl final). The mixture was laid on top of a sucrose cushion (1 ml 20 mM Tris/HCl, pH 7,4/200 mM sucrose, 100μl 20 mM Tris/HCl, pH 7,4/1 M sucrose), centrifuged for 45 min at 50000 × g at 4°C and liposomes were collected from the bottom. Fractions were subjected to SDS-PAGE and bound protein analyzed by phosphor-imaging.

#### Data Processing

The apparent dissociation constant for the FAT blot assay was determined according to

Binding % = 100% * [Lipids]/(K_D(app) _+ [Lipids])

and the least square fit analysis was performed with Sigma Plot 7 (SPSS Inc.).

## Abbreviations

DGDG, digalactosyldiacylglycerol; LPA, lipid A; MGDG, monogalactosyldiacylglycerol; SQDG, sulfoquinovosyldiacylglycerol; PC, phosphatidylcholine; pFNR, precursor of the ferredoxin NADP reductase; PG, phosphatidylglycerol; PI, phosphatidylinositol; pSSU, precursor for small subunit of ribulose-1,5-bisphosphate carboxylase/oxygenase

## Authors' contributions

ES, WL and JMS designed the experimental strategy; TW, RM and JMS conducted the experiments; and ES, TW and RM were involved in the data analysis and their processing. ES drafted the manuscript, which was subsequently finalized by WL and JMS. All authors approved the final manuscript.
